# In Vitro versus In Vivo Phase Instability of Zirconia-Toughened Alumina Femoral Heads: A Critical Comparative Assessment

**DOI:** 10.3390/ma10050466

**Published:** 2017-04-28

**Authors:** Giuseppe Pezzotti, Saverio Affatato, Alfredo Rondinella, Makiko Yorifuji, Elia Marin, Wenliang Zhu, Bryan McEntire, Sonny B. Bal, Kengo Yamamoto

**Affiliations:** 1 Ceramic Physics Laboratory, Kyoto Institute of Technology, Kyoto 606-8585, Japan; al.rondinella@gmail.com (A.R.); n14i0002@kit.ac.jp (E.M.); 2 Department of Orthopaedic Surgery, Tokyo Medical University, Tokyo 160-0023, Japan; yorifujiko@gmail.com (M.Y.); kyamamoto@pf6.so-net.ne.jp (K.Y.); 3 Medical Technology Laboratory, Rizzoli Orthopaedic Institute, Bologna 40136, Italy; affatato@tecno.ior.it; 4 Department of Medical Engineering for Treatment of Bone and Joint Disorders, Osaka University, Osaka 565-0854, Japan; wenlzhu@ort.med.osaka-u.ac.jp; 5 Amedica Corporation, Salt Lake City, UT 84119, USA; BMcEntire@amedica.com (B.M.); SBal@amedica.com (S.B.B.); 6 Department of Orthopaedic Surgery, University of Missouri, Columbia, MO 65212, USA

**Keywords:** ZTA, femoral head, Raman spectroscopy, in vivo, in vitro, hydrothermal aging

## Abstract

A clear discrepancy between predicted in vitro and actual in vivo surface phase stability of BIOLOX^®^*delta* zirconia-toughened alumina (ZTA) femoral heads has been demonstrated by several independent research groups. Data from retrievals challenge the validity of the standard method currently utilized in evaluating surface stability and raise a series of important questions: (1) Why do in vitro hydrothermal aging treatments conspicuously fail to model actual results from the in vivo environment? (2) What is the preponderant microscopic phenomenon triggering the accelerated transformation in vivo? (3) Ultimately, what revisions of the current in vitro standard are needed in order to obtain consistent predictions of ZTA transformation kinetics in vivo? Reported in this paper is a new in toto method for visualizing the surface stability of femoral heads. It is based on CAD-assisted Raman spectroscopy to quantitatively assess the phase transformation observed in ZTA retrievals. Using a series of independent analytical probes, an evaluation of the microscopic mechanisms responsible for the polymorphic transformation is also provided. An outline is given of the possible ways in which the current hydrothermal simulation standard for artificial joints can be improved in an attempt to reduce the gap between in vitro simulation and reality.

## 1. Introduction

A common tactic used in the marketing of highly technical products is the reduction of their message to its simplest possible terms—one that can be easily understood and remembered by end-customers. This strategy is also used in marketing of bioceramic medical devices. Instead of presenting detailed scientific arguments on the material’s strengths and weaknesses, the catch-phrase often used in marketing literature has been “bioceramics are inert” [1,2,3]. However, scientific discovery is always multifaceted; and it can hardly be expressed by a single phrase. In this context, the use of this statement for zirconia-toughened alumina (ZTA) might be interpreted as indicating that the phase composition of this composite remains unchanged during its extended in vivo service. The current “gold standard” for ZTA materials is BIOLOX^®^*delta*. Its manufacturer, CeramTec (Plochingen, Germany), has consistently stated that the material is bioinert. Moreover, at the time of its initial market release, they explicitly claimed that the material was also fully phase stable [4,5]. It was only after publication of experimental evidence demonstrating that autoclaved BIOLOX^®^*delta* femoral heads underwent consistent polymorphic transformation [6,7] that the manufacturer conceded that phase instability was a reality. However, based on their extrapolation of hydrothermal conditions to homeostatic temperature, they argued that it would take several hundred years of in vivo use for any appreciable transformation to be deleterious [8]. To end this diatribe, French and Japanese research groups (in cooperation with CeramTec) published a study in which the in vitro hydrothermal behavior of BIOLOX^®^*delta* was finally rationalized [9]. However, several independent research groups have subsequently reported that short- and mid-term ZTA femoral head retrievals exhibited significant amounts of unexpected polymorphic transformation [10,11,12,13,14,15]. In each study, the amount of metastable tetragonal zirconia (*t*-ZrO_2_) transformed to its stable monoclinic form (*m*-ZrO_2_) was substantially higher than predicted by thermodynamic extrapolations of the in vitro hydrothermal model [9]. This experimental evidence raises a serious concern with respect to the validity of the ASTM standardized in vitro model [16]. In its current form, the model fails to predict the in vivo surface phase instability of hip joint components made from ZTA. Recently, we have reported that short-term surface aging and degradation of the ceramic femoral heads might be induced by metal contamination in femoral heads, a common phenomenon in hip arthroplasty [15].

This paper seeks to further resolve this controversy. To do so, a series of BIOLOX^®^*delta* retrievals were extensively characterized to determine the effect of the human body environment on the rate of the *t*→*m*-ZrO_2_ transformation. In vitro experiments were then performed to assess the fundamental role of the initial *m*-ZrO_2_ content on the kinetics of the transformation. Comparisons were made between the in vitro data (normalized to in vivo lifetimes according to the ASTM standard [16]) and the retrievals. To reconcile the large observed discrepancies between the in vitro and in vivo data, additional factors that may have accelerated the transformation were also investigated. These potential “triggers” included the size of the zirconia domains, mechanical stress, and autocatalytic surface reactions from metallic contaminants. The findings suggest that the polymorphic transformation of ZrO_2_ is significantly more complex than previously assumed. Indeed, it cannot be understood or rationalized under the sole assumption of hydrothermal activation of the ZTA’s surface. The current experimental findings call for a prompt revision of the international standards that regulate the market release of biomedical ZTA for artificial joints.

## 2. Materials and Methods

The investigated samples were ZTA femoral heads (BIOLOX^®^*delta* CeramTec AG, Plochingen, Germany). This material was clinically introduced in Europe in 2003, in the US in 2004, and in Japan in 2011. Its composition consists of an alumina matrix (Al_2_O_3_; 82 vol.%) reinforced by yttria-stabilized zirconia (Y-TZP; 17 vol.%), chromium oxide (Cr_2_O_3_; 0.5 vol.%), and strontium oxide (SrO; 0.5 vol.%). The fraction of yttrium oxide (Y_2_O_3_) added to the zirconia phase is ~1.3 mol.%, which partially stabilizes it in its tetragonal polymorph (i.e., Y-TZP). The two minor additives, Cr_2_O_3_ and SrO, were incorporated into the alumina matrix in order to increase the material’s hardness [13]. Thirty-two new ZTA femoral heads were tested, which were released to the market over a period of ~10 years (i.e., 2005–2015). This set of samples included 6, 8, 5, and 3 heads manufactured in 2009, 2011, 2014, and 2015, respectively. An additional 10 heads were all manufactured before 2009. As discussed in detail later, the mean sizes of alumina and zirconia grains in these ZTA heads fluctuated according to individual components; but they were all on the order of a single micron and hundreds of nanometers, respectively.

The ASTM standard [16] hydrothermal aging test was conducted on 28 mm-diameter ZTA head specimens (*n* = 3) using a high-pressure steam sterilizer (TOMY SX-300, Tomy Seiko, Co., Tokyo, Japan). The ZTA samples were exposed to 134 °C water steam under a pressure of 2 bar for up to 20 h. As theoretically defined in ASTM methodology, 1 h of aging under the above-mentioned conditions corresponds approximately to two years of in vivo exposure [16]. After sequential intervals of 2.5 h in the autoclave, the transformed percentages of monoclinic zirconia were non-destructively measured using confocal Raman spectroscopy. Tests were also performed at different autoclave temperatures (121~134 °C) on as-received femoral heads with and without laboratory-induced metal stains (Fe, CoCr, Ti) on their surfaces. Hardness values on some of the as-received femoral heads were obtained using a Vickers pyramidal diamond indenter (load 5 Kgf) prior to the autoclave tests.

Twenty-two short- to mid-term BIOLOX^®^*delta* ZTA retrievals were analyzed, including 9 ceramic-on-polyethylene (CoP) and 13 ceramic-on-ceramic (CoC) samples, explanted after in vivo periods spanning ~2 months to ~9 years. Revisions were performed for infection (5 cases), aseptic loosening (2 cases), dislocation (10 cases), stem loosening (1 case), septic mobilization (2 cases), aseptic mobilization (1 case), and thigh pain (1 case). Fractured heads were not included in this study. Table 1 lists all of the retrievals along with their respective year of manufacture, their in vivo service lifetimes, and the reasons for their removal during revision surgery.

The percentages of transformed monoclinic volume fraction, *V_m_*, in as-received, autoclaved, and retrieved ZTA samples were measured by means of a confocal Raman spectrometer. The excitation source was a 488 nm Ar-ion laser (GLG3103, Showa Optronics Co., Ltd., Tokyo, Japan) yielding a power of approximately 35 mW on the sample surfaces. Raman spectra were detected using high-resolution spectrometers (MS3504i, SOL instruments Ltd., Minsk, Republic of Belarus; or T-64000, Horiba/Jobin-Yvon, Kyoto, Japan). A confocal configuration of the optical probe was adopted throughout the experiments, which corresponded to a ×100 objective (numerical aperture, focal length, and pinhole diameter fixed as 0.8, 3.4 mm, and 100 μm, respectively). Individual Raman spectra were typically collected in five-second intervals. The focal spot size at the head surfaces was ~1 μm. A diameter of 100 μm was set for the pinhole aperture in order to cut the light from the sub-surface region, thus acquiring spectra from the first five microns below the surface. Spectra were acquired in backscattering geometry with a spectral resolution of ~0.5 cm^−1^ achieved by a 2400 grooves/mm grating. The recorded spectra were averaged over three successive measurements. An in-plane sampling of 2.5 μm lateral steps was applied and a spectral map of 50 × 50 μm^2^ in dimensions was collected (for a total of 1323 spectra per each map). Deconvolution of all the recorded spectra into sub-bands was performed according to mixed Gaussian/Lorentzian curves, which were adjusted to fit the experimental spectra using commercially available computational software (LabSpec 3, HORIBA Jobin-Yvon SAS, Lille, France). The band intensities were calculated after spectral fitting, and an estimate of the *V_m_* values was made according to the Katagiri’s equation [7,17]. A two-tailed Student’s *t* test was performed with the aid of Graphpad Prism software, version 6.05 (GraphPad software, Inc., San Diego, CA, USA) to test for statistical significance of *V_m_* contents at different locations on the ZTA femoral heads. Differences were considered to be significant at the *p* < 0.05 level.

Selected ZTA retrievals were scanned in toto with respect to their topological and spectroscopic response (cf. samples labeled with an asterisk in Table 1). In this procedure, a configuration was built using computer aided design software (CAD). A polar grid to cover the entire dome of the studied heads was developed as shown in a previous publication [18]. The dimension of each element was set to generate a fine grid of 16 meridians, while a separate 2 mm diameter circular element was selected on the top of the retrieval. For each portion of the surface, micrographs were taken by mean of a 3D laser-scanning microscope (VK-X200K series, Keyence, Osaka, Japan) using both 10× and 150× objective lenses with a numerical aperture of 0.95. The instrument’s software provided maps, profiles, average surface roughness (*R*_a_), and statistical distributions of roughness in accordance with ISO 4287:1997. Samples were subsequently placed on an *x*-*y* axes motorized stage (lateral resolution of 0.1 µm) and scanned with the Raman probe for the purpose of collecting in toto maps of phase transformation. An algorithm written using RStudio (RStudio, Team 2015) allowed automatic calculations which efficiently handled a large number of spectra with respect to the intensity and spectral position of selected bands. Residual stresses in the constituent phases of the ZTA heads were computed from spectral shifts according to piezo-spectroscopic coefficients previously calibrated for the bands at 145 for *t*-ZrO_2_, 178 for *m*-ZrO_2_, and 415 for Al_2_O_3_ cm^−1^ as −0.6, −0.9, and −0.76 cm^−1^/GPa, respectively [19,20]. Negative piezo-spectroscopic coefficients represent compressive stress fields which induce spectral shifts of the Raman bands toward higher frequencies and vice versa for tensile stresses.

The confocal probe was eventually shifted toward the in-depth direction of the ZTA samples to non-destructively acquire Raman sub-surface maps. Lastly, a 3D model for each retrieval was acquired by means of CAD software (SolidWorks, Waltham, MA, USA); and a combined procedure was automatically run to non-destructively obtain an overall quality assessment of each retrieval for roughness, phase transformation, and residual stress with microscopic probes used for screening at the macroscopic scale.

Cathodoluminescence (CL) spectra were collected using a field-emission gun scanning electron microscope (FEG-SEM, SE-4300, Hitachi Co., Tokyo, Japan) as an excitation source. Electron irradiation was made with an acceleration voltage of 5 kV. Preliminary calibrations proved that the selected acceleration voltage was below the threshold for perturbation of the stoichiometric structure of the material by electron beam impingement. The CL device consisted of an ellipsoidal mirror and a bundle of optical fibers, which enabled the collection and transmission of the CL emission into a highly spectrally resolved monochromator (Triax 320, Jobin Yvon/Horiba Group, Tokyo, Japan). A 150 g/mm grating was used throughout the experiments. A liquid nitrogen-cooled 1024 × 256 pixels CCD camera was employed to analyze the CL emission of the material. Spectral lines were analyzed with the aid of a commercially available software package (LabSpec 4.02, Horiba/Jobin-Yvon, Kyoto, Japan). Spatially resolved CL maps were collected with a lateral step of 50 nm. Spectral fitting was conducted upon deconvolution of the spectra into Gaussian-Lorentzian curves and after subtracting a linear baseline. The CL probe size was preliminarily calibrated and found to be on the order of 68 and 280 nm in depth and in plane, respectively. All mathematical procedures for modeling the CL intensity emission were carried out with the aid of commercially available computational software (Mathematica 5.2, Wolfram Research Inc., Champaign, IL, USA).

## 3. Results and Discussion

### 3.1. Discrepancies between In Vitro and In Vivo Phase Stability

**Figure 1:**
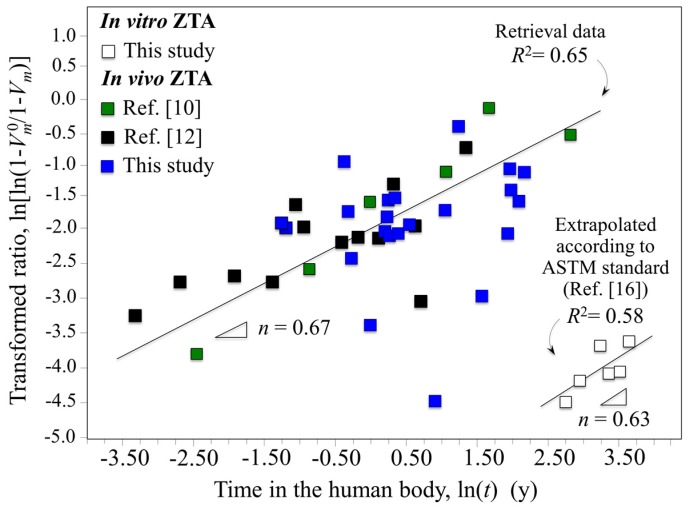
Log–log plot of time vs. monoclinic transformation ratio comparing retrieved and in vitro hydrothermal tested femoral heads.

Data for retrieved and in vitro hydrothermal tested femoral heads are compared in Figure 1 using a log–log plot with time on abscissa and the monoclinic transformation ratio on the vertical axis (i.e., ln(ln((1 − *V_m_*^0^)/(1 − *V_m_*))) vs. ln(*t*); *V_m_*^0^ is the initial monoclinic phase fraction prior to hydrothermal aging). Since *V_m_*^0^ values for individual retrievals were unknown prior to their implantation, the average monoclinic fractions measured on the non-wear/non-contact zones were assumed to be *V_m_*^0^ values. In the simulated plot, in vitro data were converted to in vivo lifetimes in accordance with ASTM F2345-03 (i.e., 2.5 h of in vitro aging at 134 °C represents ~5 years in vivo at homeostatic temperature) [16]. This standard is based on the prevailing theory governing the kinetics of the phase transformation known as the Mehl–Avrami–Johnson (MAJ) equation [7,21,22,23]. The linear dependence of the phase transformation ratio (vertical axis) allows the determination of the Avrami exponent, *n*, according to the following relation [21]:

(1) $$\ln\left[\ln\left(\frac{1-{V_{m}}^{0}}{1-V_{m}}\right)\right]=nlnb+nlnt,$$

where *b* is a parameter that represents the temperature dependence of the aging effect, *t* is the aging duration, and *n* is a time exponent (which is independent of temperature). The *n* exponent can be derived directly from the logarithmic form of Equation (1) by best-fitting of the linear regression line as those shown in Figure 1. The *n* value intrinsically reflects rates for both nucleation and growth of monoclinic ZrO_2_ domains. Note here that wear caused by any abnormal activity, acidosis, and/or overloading due to abnormal body weight of the patients were not considered. These factors may result in a deviation of the obtained data from the linear plot. However, it is yet remarkable that a linear plot could be drawn with a reasonable approximation and a statistically larger number of investigated retrievals including other published data could increase the reliability of the plot. Therefore, when displayed on this log–log scale, ZTA retrieval data from different studies were also rationalized on the same plot regardless of the type of implant (i.e., CoC or CoP) [10,12]. Experimental data from previous studies on yttria-stabilized zirconia demonstrated that *n* exponents ranged between 0.5 and 4 [21,22]. For *n* values close to 4, the preponderant contributor to the transformation kinetics is the growth of pre-existing monoclinic nuclei (*V_m_*^0^); whereas small *n* values are representative of high nucleation rates. Because the data for the in vitro simulated and in vivo retrieved ZTA samples of Figure 1 have *n* exponents in the low range (i.e., *n* = 0.67 (*R*^2^ = 0.65) and 0.63 (*R*^2^ = 0.58), respectively), the transformation kinetics for both types of ZTA materials are dominated by the formation of monoclinic nuclei. Moreover, an Avrami exponent of *n* < 1 suggests that the overall growth mechanism is slow and the subsurface increase in the monoclinic phase is delayed [21]. In spite of the observed consistency of the Avrami slopes of Figure 1, there is a discrepancy between in vitro and in vivo plots. Utilizing the ASTM standard, the extrapolated in vitro simulations completely failed to predict the amount of transformation occurring in vivo by several orders of magnitude. The experimental data for the retrievals clearly conflict with the manufacturer’s statement: [8] “the accelerated ageing was simulated by 5 h and 100 h treatment in autoclaving conditions which is equivalent to 10 years and 200 years (!) in vivo.” Indeed, the observed transformation rates in vivo were ~10^3^ faster than predicted by ASTM standard (i.e., 1 h in vitro appears to correspond to ~18 h in vivo). These results suggest that in vitro low-temperature simulation studies on the aging of ZTA ceramics have serious limitations when extrapolated to in vivo conditions. Furthermore, the current ASTM standard is inadequate in predicting transformation lifetimes for the in vivo environment. Possible origins for the striking failure of the standard could be micromechanical and/or chemical; but both point to the possibility of extrinsic factors responsible for the phase transformation in vivo. These factors will be experimentally investigated and discussed in subsequent subsections.

Undoubtedly, the Al_2_O_3_ matrix within the ZTA microstructure is at least partly responsible for the small *n* exponent. The transformation process is limited to isolated ZrO_2_ grains due to the constraining effects of the surrounding Al_2_O_3_ matrix. Pecharromán et al. [24] recommended an upper limit of 16 vol.% (~22 wt.%) for zirconia concentration in an alumina matrix in order to avoid subsurface spreading of monoclinic domains. This ZrO_2_ concentration corresponds to the theoretical percolation threshold. In other words, when the Al_2_O_3_ content is below 74 vol.%, the material’s stability can abruptly deteriorate due to the statistical connectivity of adjacent ZrO_2_ grains. Note that the ZrO_2_ fraction in the studied ZTA femoral heads is exactly at the percolation limit. The compositional standard for zirconia-based ceramics, ISO 13356, allows an initial *m*-ZrO_2_ fraction of up to 20 vol.%. Because of this, CeramTec’s technical literature states that [8]: “the specific composition of BIOLOX^®^*delta* provides inherent protection against improper phase transformation.” However, this statement is at odds with the aforementioned scientific literature which demonstrated that ZrO_2_ contents at or above the percolation limit are inherently unstable and risk accelerated surface and sub-surface transformation.

### 3.2. The Effect of the Initial Monoclinic Fraction

**Figure 2:**
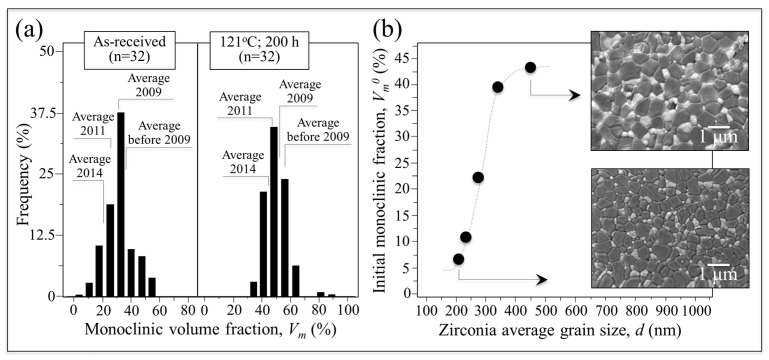
(**a**) Histograms showing the range of initial monoclinic fractions for BIOLOX^®^*delta* components released through the years; and (**b**) relationship between average size of the zirconia domains in the BIOLOX^®^*delta* microstructure and their initial amounts of monoclinic fraction, *V_m_*^0^.

The large range of initial monoclinic fractions for BIOLOX^®^*delta* components released through the years is provided in the histograms of Figure 2a. These were collected on 32 ZTA as-received femoral heads manufactured during the decade 2005–2015. The histogram in the left-hand plot shows the percentage of heads and their average monoclinic phase fractions as a function of manufacturing date, while the right-hand histogram gives the statistical evolution of the monoclinic content after an autoclave cycle of 121 °C at adiabatic pressure. The left plot indicates that the BIOLOX^®^*delta* femoral heads released to the market contained average surface *m*-ZrO_2_ phase fractions fluctuating between 10% and 55%. Note that there is a higher average fraction for heads manufactured on or before 2009. When average data are grouped according to manufacturing year, improved manufacturing control of the *m*-ZrO_2_ phase fraction is noted in successive years. Nevertheless, the data still indicate a broad distribution of surface monoclinic contents for the marketed heads. Autoclaving the heads in a hydrothermal environment increases the monoclinic polymorph as expected (i.e., in the range of 30%~65%) while currently narrowing the width of the statistical distribution. Although differences between sets of components from specific manufacturing years were generally reduced, there were still isolated instances of monoclinic contents of between 80% and 90%. Figure 2b correlates the average size of the zirconia domains in the BIOLOX^®^*delta* microstructure with their initial amounts of monoclinic fraction, *V_m_*^0^. The size of zirconia domains, *d*, which was obtained by a random-line intercept method from scanning electron micrographs, fluctuated between 200 and 500 nm. Remarkably, the fraction of transformed zirconia in the as-received components, *V_m_*^0^, increased sharply with increasing domain size, but tended to saturate above 500 nm. The average size of the alumina grains (not shown) fluctuated between 500 and 800 nm. In other words, the observed increase in *V_m_*^0^ clearly corresponded to a coarsening of the ZTA microstructure. There are multiple potential reasons for microstructural coarsening in ZTA sintered bodies including variations in the particle size of the raw materials, thermal fluctuations during sintering, and the homogeneity of the sintering additives.

**Figure 3:**
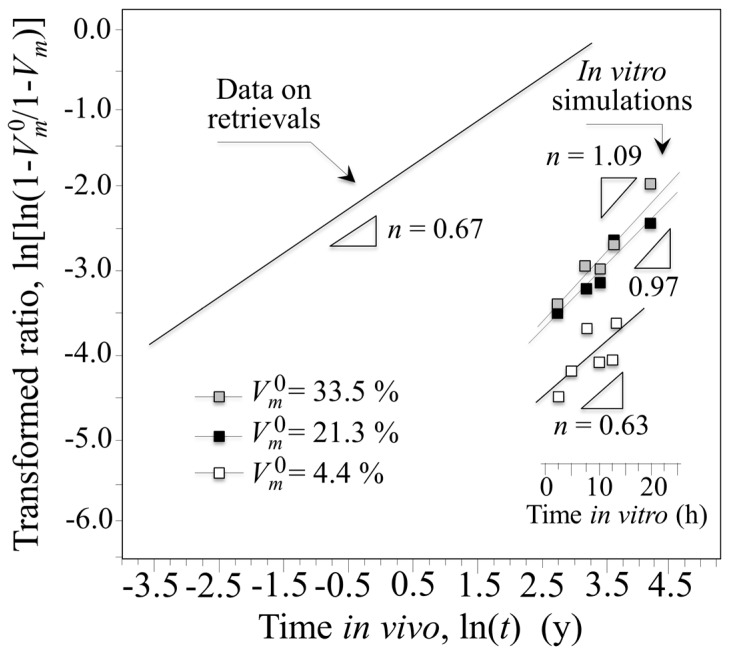
Monoclinic fractions for the retrievals and for in vitro hydrothermally treated heads at 134 °C as a function of time and extrapolated in vivo lifetime according to ASTM F2345-03.

Monoclinic fractions for the retrievals and for in vitro hydrothermally treated heads at 134 °C as a function of time are shown in Figure 3. While the tradition MAJ equation suggests only an additive role for the initial monoclinic content, it was noted that the transformation ratio appeared to also correlate with apparent activation energy. To rationalize this effect, the traditional MAJ equation was modified to show that the Avrami exponent, *n*, has a dependence on the temperature. Rearranging from Equation (1) gives the following relation: [25]

(2) $$\;\ln\left[\ln\left(\frac{1-{V_{m}}^{0}}{1-V_{m}}\right)\right]=n\left(lnb_{0}+lnt\right)-\frac{nQ}{RT},$$

where *b*_0_ is a material constant, *Q* is the activation energy for phase transformation during environmental ageing, *R* is the universal gas constant, and *T* is the absolute temperature. Accordingly, the slope of a plot of $\ln\left[\ln\left(\frac{1-{V_{m}}^{0}}{1-V_{m}}\right)\right]$ vs. *lnt* at constant temperature becomes *n*, while the slope of a plot of $\ln\left[\ln\left(\frac{1-{V_{m}}^{0}}{1-V_{m}}\right)\right]$ vs. $\frac{n\left(T\right)}{RT}$ is intrinsic activation energy. Note that the formalism of Equation (2) predicts the existence of an intrinsic value for the activation energy, *Q*, but allows variations of the apparent activation energy value, *Q*_app_, since *n* is also a function of temperature. The plots of Figure 3, which give the phase-transformation ratio as a function of ln*t* for components with different initial monoclinic ratios (i.e., different zirconia domain size; cf. Figure 2b) at the constant temperature of 134 °C can now be rationalized according to their different Avrami slopes, *n*. However, the dominant kinetic mechanism is still the nucleation rate of monoclinic domains (*n* ≤ 1), but there is an increasing contribution from the growth of the formed nuclei as well.

When the plot is extrapolated to in vivo lifetimes according to ASTM F2345-03 and drawn vs. the in vivo recorded average dependence from Figure 1, a role can clearly be envisaged for the dependence of transformation kinetics on *V_m_*^0^ (or *d*). While this can partly explain the scatter of in vivo data in Figure 1, it cannot fully justify the gap between in vitro and in vivo transformation kinetics. Accordingly, there appears to be an additional trigger(s) for the in vitro/in vivo experimental discrepancy.

### 3.3. The Stress Trigger

**Figure 4:**
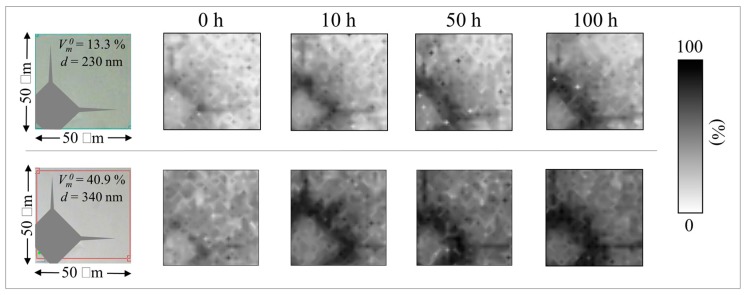
Transformation kinetics for in vitro hydrothermal aging experiments using BIOLOX^®^*delta* ZTA heads printed with 5 kgf Vickers indenter on their as-received surfaces. Initial monoclinic fractions before indentation and the sizes of the indentation print are given in inset.

The effects of mechanical stress induced by in vivo wear, impact, or shock are not included in the standard in vitro simulation test. This may be one reason why simulated results are inadequate in predicting in vivo ZTA transformation kinetics. Perrichon et al. [26] recently suggested an improved protocol for in vitro aging of metastable ceramics which included the effect of surface stresses in evaluating their hydrothermal stability. Their protocol certainly represents an improvement over the current standard, although it is not clear whether the presence of mechanical stress is the main trigger for enhanced in vivo transformation reported in Figure 1. In order to shed light on the contribution of mechanical stress on the transformation kinetics, in vitro hydrothermal aging experiments using BIOLOX^®^*delta* ZTA heads were repeated with 5 kgf Vickers indents applied to their as-received surfaces. Figure 4 shows the results of these experiments. They were conducted on two individual ZTA heads whose microstructures consisted of ZrO_2_ domains of different sizes and, accordingly, different initial amounts of monoclinic phase contents, *V_m_*^0^. These heads were subsequently autoclaved (cf. labels). Maps of their monoclinic fraction with increasing autoclave exposure (up to 100 h at 134 °C) clearly revealed an alteration of the phase-transformation kinetics. Zones around the imprints generally demonstrated accelerated transformation rates; and these alterations were clearly more pronounced on the component with the coarse microstructure and higher initial *V_m_*^0^.

**Figure 5:**
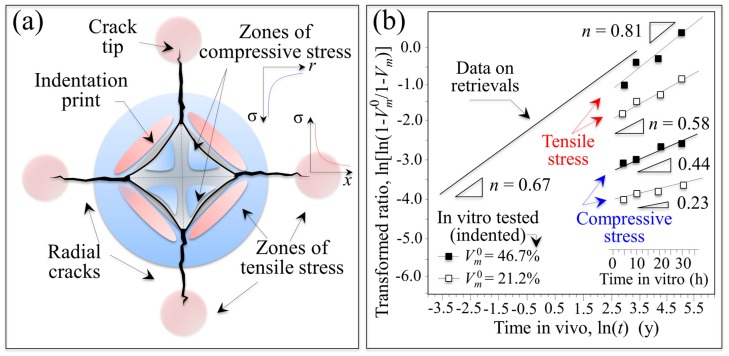
(**a**) Schematic of a Vickers indentation print and the associated residual stress field ccording to Yoffe’s theory [29]. An indent introduces tensile (red) and compressive (blue) residual stress fields in different zones on the material’s surface; (**b**) Plots of the phase-transformation parameter, ln(ln((1 − *V_m_*^0^)/(1 − *V_m_*))), in both tensile and compressive stress zones around the indents as a function of both autoclave time and extrapolated in vivo lifetime according to ASTM F2345-03.

Residual stress fields around the indentations were studied using protocols from previously published reports in order to quantify differences in transformation rates [27,28]. According to Yoffe’s theory [29] (schematically shown in Figure 5a), an indent introduces positive and negative residual stress fields in different zones on the material’s surface. Tensile stress fields are created due to the generation of small microcracks in the vicinity of the outside edges of the indent (i.e., hoop stresses on the order of few hundreds MPa) [30,31] and at the tip of the main radial microcracks propagated from the imprint corners (i.e., red areas in Figure 5a). However, compressive radial stresses of several GPa remain within the imprint in areas close to the indentation edges (e.g., blue areas in Figure 5a) and their magnitude decreases with increasing distance from the print center. In these areas, where plastic deformation is less pronounced, elastic residual stress fields can be measured [27]. In non-transforming ceramics, the elastic residual stress fields obey Yoffe’s formalism to a high degree of precision [27]. Because ZTA is prone to polymorphic transformation, the Yoffe stress is instantaneously altered by an overlapping stress field induced by the volume expansion associated with the transformation of the zirconia dispersoids [28]. A comparison between the Raman maps in Figure 4 and the schematic of Figure 5a reveals that the zones under residual stress are tensile in nature. These tensile zones are transformed the most; and they will continue to transform with increasing autoclave time.

Plots are given in Figure 5b of the phase-transformation parameter, ln(ln((1 − *V_m_*^0^)/(1 − *V_m_*))), in both tensile and compressive stress zones around the indents as a function of both autoclave time and extrapolated in vivo lifetime according to ASTM F2345-03. The related Avrami slopes, *n*, are given in the inset. The transformation fractions were quite different for regions affected by tensile and compressive stresses; whereas the monoclinic fractions developed after autoclaving were significantly influenced by the initial monoclinic fraction, *V_m_*^0^ (i.e., higher *V_m_*^0^ resulted in a greater amount of *t*→*m* ZrO_2_ transformation). On the one hand, tensile residual stresses greatly enhanced the transformation rate while generating a relatively high Avrami slope. On the other hand, the effect of compressive stresses was less significant in terms of enhanced transformation and resulted in lower Avrami exponents, *n*, when compared to non-indented samples. In terms of nucleation and growth, it appears that nucleation controlled the surface transformation kinetics regardless of the nature of the residual stress. Tensile stresses accelerated the transformation kinetics with increased contributions from nuclei growth (i.e., a larger Avrami exponent); and compressive stresses slowed the transformation. Nuclei formation was predominant (as testified by a reduced Avrami slope) when compared to unstressed samples.

A comparison with the retrievals data (cf. Figure 5b) suggests that tensile residual stresses in components with an initially high fraction of monoclinic phase may be a powerful trigger of the polymorphic transformation in vivo. However, tensile stresses alter the way in which polymorphic transformation proceeds, with enhanced contributions from nuclei growth (i.e., higher Avrami slope). In principle, tensile residual stresses justify the faster transformation rates observed in vivo with respect to the static hydrothermal stress in vitro. However, despite being larger in magnitude than the tensile ones, compressive stresses were less effective in raising the in vitro plot in Figure 5b to the levels observed on retrievals. The key point is this: Independent of whether the heads came from CoC or CoP couples, they were absent of any damage or microcracks similarly induced on the indented samples. This finding is in line with previous reports [12,15]. In addition, hard-on-hard impingement or third-body wear usually introduce compressive rather than tensile stresses in ceramic couples [32]. Moreover, it is not immediately obvious how the presence of a softer sliding counterpart in CoP couples could cause tensile residual stresses of the relatively high magnitude necessary to accelerate the polymorphic transformation. These arguments point to the possible presence of an additional trigger, perhaps of chemical nature, which is discussed in the next subsection.

### 3.4. The Chemical Trigger

**Figure 6:**
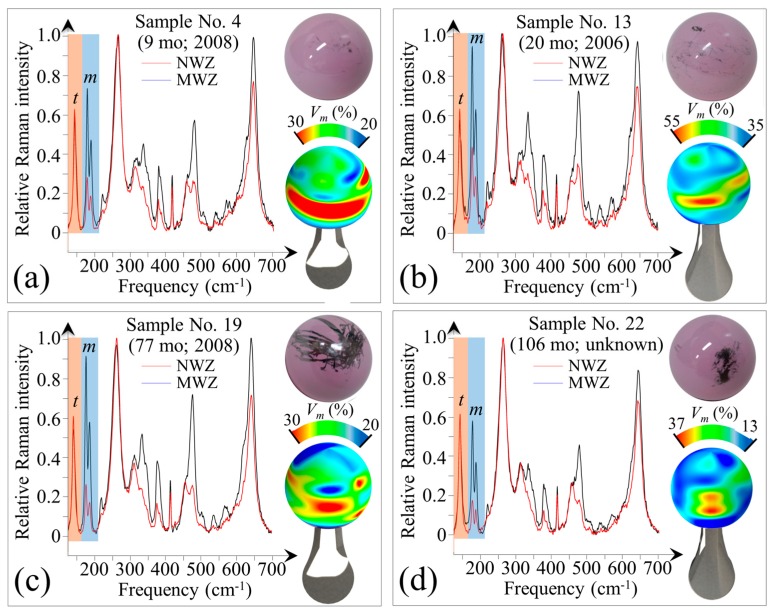
Pictures of four selected retrievals and in toto Raman maps of their surfaces: No. 4 (**a**); No. 13 (**b**); No. 19 (**c**); and No. 22 (**d**) (cf. labels in the inset and Table 1); average Raman spectra for MWZ and NWZ including bands for the tetragonal (*t*) and monoclinic (*m*) polymorphs are also shown for each retrieval.

A program of in toto Raman screening of some of the retrieved femoral heads was conducted in an effort to uncover possible additional triggers for the in vivo polymorphic transformation. Figure 6a–d shows pictures of four selected retrievals and in toto Raman maps of their surfaces (cf. labels in the inset and Table 1). Average Raman spectra for the main-wear zones (MWZ) and non-wear zones (NWZ) were compiled including bands for the tetragonal and monoclinic polymorphs, labeled as *t* and *m* in Figure 6a–d, respectively. All of these samples showed metal contamination both in their MWZ and NWZ. The samples in Figure 6a,b (No. 4 and No. 13 in Table 1, respectively) had weak metal contamination with only small random patches and patterned coverage (small straight lines evenly distributed) along with a small solid patch on the upper hemisphere, respectively. The samples in Figure 6c,d (No. 19 and No. 22 in Table 1, respectively) both showed metal contamination with a number of random stripes, a large solid patch, and an extensive massive patch. The former two metal-stains are due to partial subluxation and impingement of the metal cup on the ceramic head. The later stain is a consequence of repeated prosthetic dislocations during surgical implantation. There were clear correlations between the topological stains and the monoclinic phase contents of these retrievals. This can be easily visualized in Figure 6c,d. However, high amounts of *m*-ZrO_2_ also existed on apparently “clean” MWZ surfaces (i.e., non-stained, cf. Figure 6a) as well as in NWZ areas with weak metal pits.

**Figure 7:**
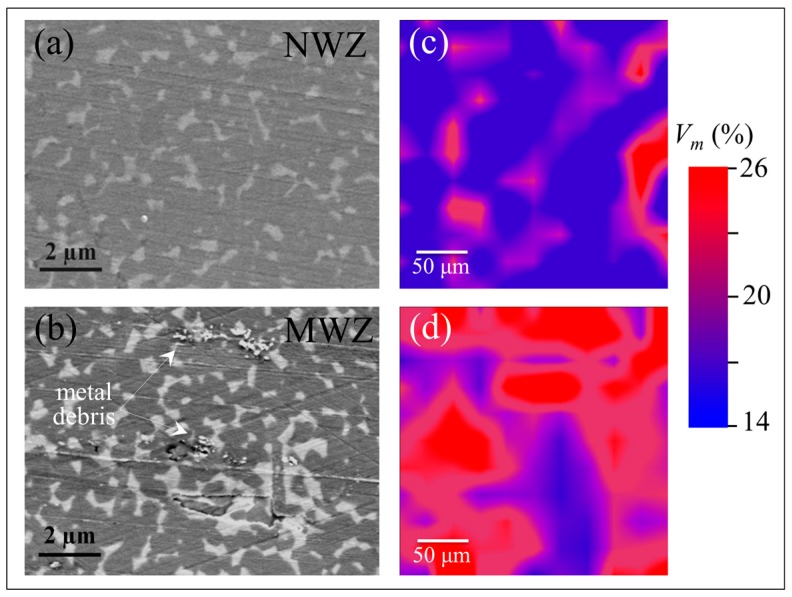
Scanning electron micrographs of the: NWZ (**a**); and MWZ (**b**); and Raman spectroscopic analyses in the corresponding areas ((**c**,**d**), respectively); note the enhanced transformation in correspondence of metal debris and the very low amount of wear in both NWZ and MWZ, as evidenced by the presence of detectable machining lines on the ceramic surface.

As shown in Figure 7, concurrent scanning electron and Raman analyses of the NWZ (Figure 7a) and MWZ (Figure 7b) areas showed that the presence of microscopic metal debris greatly enhanced the transformation (cf. NWZ and MWZ in Figure 7c,d). Note that there is a very low amount of wear in the NWZ (Figure 7a) and MWZ (Figure 7b) as evidenced by the presence of detectable machining lines on the ceramic surface. While it is possible that some retrievals may have already had high amounts of transformation prior to implantation due to their early year of manufacture (cf. labels for Nos. 4, 13, and 19 in Figure 6), the preponderance of the evidence suggests that chemistry plays a critical role in the transformation. This conclusion is not only evident in the vicinity of heavy metal stains and patches, but also by the presence of diffuse metal ions.

**Figure 8:**
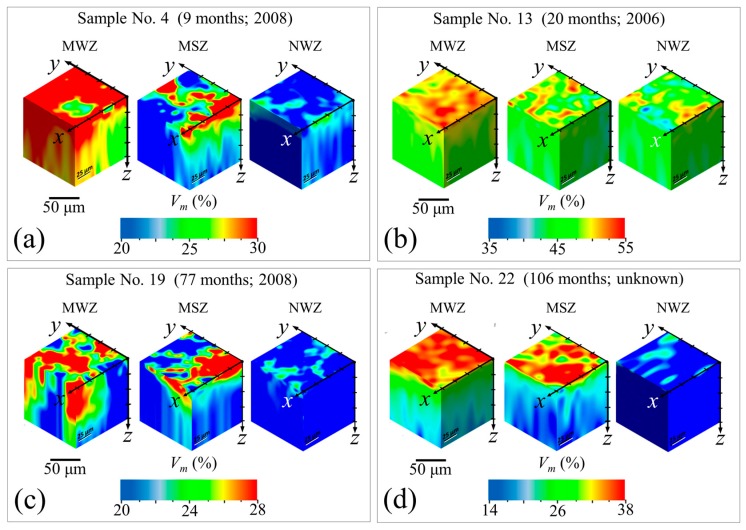
Maps for the in-depth transformation on the metal-stained MWZ, metal-stained NWZ (labeled as metal-stained zone, MSZ), and non-stained NWZ of Samples: No. 4 (**a**); No. 13 (**b**); No. 19 (**c**); and No. 22 (**d**) (cf. Table 1).

In-depth confocal Raman measurements were collected on the samples shown in Figure 6 in order to non-destructively trace the transformation profile into the head’s sub-surface. Figure 8a–d shows selected maps for the in-depth transformation on the metal-stained MWZ, metal-stained NWZ (labeled as metal-stained zone, MSZ), and non-stained NWZ of Samples No. 4, 13, 19, and 22.

In general, there was a gradual decrease in monoclinic content with increasing depth, *z*. However, the MWZs experienced relatively higher monoclinic factions at greater *z* values as compared with their respective MSZs and NWZs. This finding suggests that it is the combination of mechanical stress and metal contamination enhanced by an initially higher amount of initial monoclinic content, *V_m_*^0^ (i.e., the strong trigger) that leads to accelerated transformation (at least to 50 µm depth). Conversely, the amount of transformation for metal stained areas in the absence of surface stress is limited to a few sub-surface microns. Nevertheless, the presence of diffuse metal ions is apparently sufficient to significantly accelerate the transformation occurring at the ceramic’s surface. Because these analyses indicate that metal contamination (a common phenomenon in total hip arthroplasty) markedly affects surface metastability, an experimental in vitro program was designed and conducted to mechanistically understand and quantify the effects of this phenomenon.

**Figure 9:**
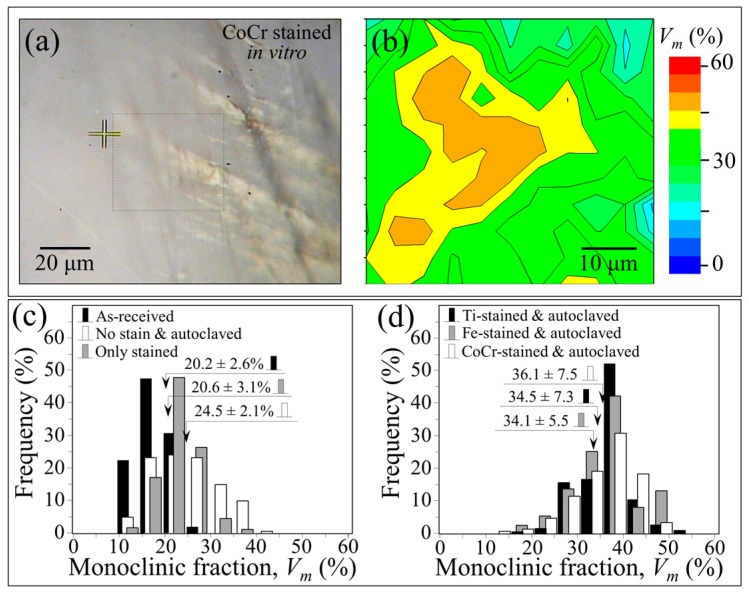
(**a**) Optical micrograph of the surface of a CoCr-stained ZTA femoral head and Raman map; and (**b**) obtained in the vicinity of the CoCr stain in correspondence of the squared inset of (**a**); (**c**) satistical histograms of monoclinic fractions for the same ZTA head in (**a**) as-received, autoclaved without staining, and CoCr-stained before autoclaving; and statistical histograms comparing monoclinic fractions after staining and autoclaving for CoCr, Fe, and Ti (**d**).

Raman maps were collected at selected regions of as-received ZTA femoral heads before and after intentionally introducing different metal stains at room temperature. The metal stains were simply applied by gently rubbing the surfaces of the femoral heads with a metal rod analogous to how chalk is used on a blackboard. These same zones were then mapped again using the Raman probe after in vitro exposure for 24 h in water vapor at 121 °C under adiabatic pressure. Figure 9a shows an optical micrograph of the surface of a CoCr-stained ZTA femoral head after this exposure. A Raman map obtained in the vicinity of the CoCr stain, which corresponds to the squared inset of Figure 9a, is shown in Figure 9b. The Raman map showed a relatively high amount of transformed *m*-ZrO_2_ adjacent to the metal stain (i.e., average value *V_m_^av^* = 36.1% ± 7.5% and maximum value *V_m_^max^* = 49.6%); whereas the same sample showed negligible changes in its monoclinic fraction after staining but before autoclaving when compared with its pristine condition (i.e., *V_m_^av^* = 20.6% ± 3.1% vs. 20.2% ± 2.6%; *V_m_^max^* = 23.3% vs. 22.3%; cf. labels in the inset to Figure 9c). Similar low *m*-ZrO_2_ values were found after autoclaving an unstained sample (Figure 9c). These results demonstrate that enhanced destabilization is mainly due to chemical effects associated with the metallic stains and not merely the result of either hydrothermal attack or mechanical stress.

Raman maps of *m*-ZrO_2_ contents were acquired for all types of stains (i.e., CoCr, Fe, and Ti) at exactly the same locations before and after staining, and again after autoclaving. Statistical histograms comparing monoclinic fractions after autoclaving are shown in Figure 9d. Identical trends were found for all of the metallic stains, leading to the following conclusions: (i) the mechanical action of room temperature staining introduced negligible amounts of polymorphic transformation in the stained zone; (ii) a short term autoclave treatment was sufficient to increase the amount of *m*-ZrO_2_ by 50% or more in the stained areas when compared to the as-received controls; and (iii) areas far away from the stains showed minimally higher amounts of *m*-ZrO_2_ after short-term autoclaving as compared to pristine samples.

**Figure 10:**
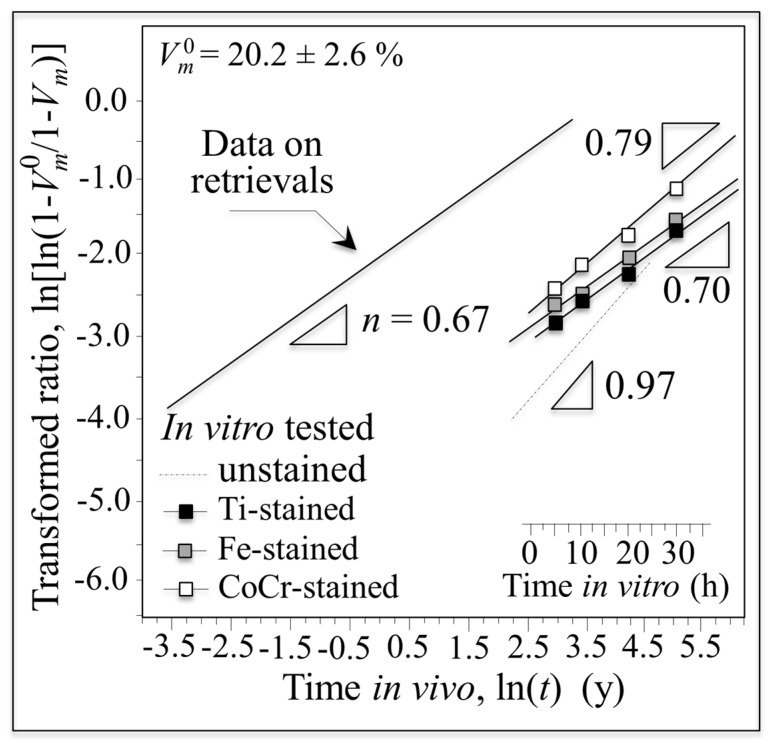
Monoclinic fractions for the retrievals and for stained and in vitro hydrothermally treated heads at 134 °C as a function of time and extrapolated in vivo lifetime according to ASTM F2345-03.

After acquiring a complete set of in vitro data at 134 °C, the exercise of plotting the phase-transformation parameter, ln(ln((1 − *V_m_*^0^)/(1 − *V_m_*))), in the metal stained zones as a function of both time in autoclave and ln*t* was repeated (Figure 10). In vitro conditions were extrapolated to in vivo lifetimes according to ASTM F2345-03. Avrami slopes, *n*, given in the inset, were similar for all the types of investigated stains and close to those recorded for retrievals. The in vitro plots for the transformed fraction on the metal-stained samples demonstrated accelerated transformation kinetics and higher Avrami slopes when compared to unstained reference sample (i.e., broken line in the plot). This indicates that metal stains significantly impact the *m*-ZrO_2_ nucleation rate, with nuclei multiplying faster in the vicinity of the stained areas.

### 3.5. Polymorphic Transformation and Its Triggers at the Molecular Scale

**Figure 11:**
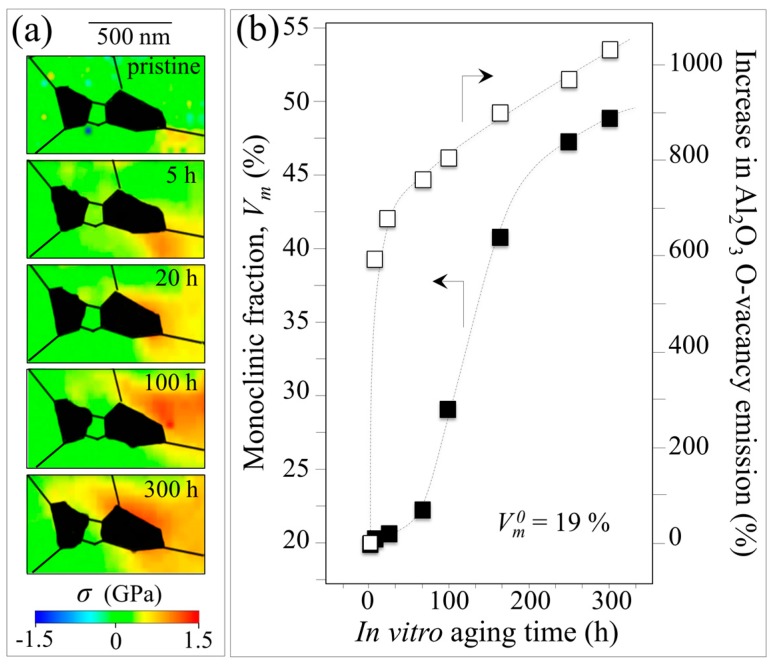
(**a**) CL assessment of the stress field, σ, in the vicinity of metastable zirconia domains in pristine BIOLOX^®^*delta* ZTA femoral heads and after they have been subjected to increasing exposures in the autoclave at 121 °C (for 5~300 h; cf. labels in inset); and (**b**) plots showing off-stoichiometric drift within the alumina matrix (i.e., the intensity of the CL emission from oxygen vacancy sites) and the amounts of polymorphic transformation by Raman spectroscopy.

The previous subsections reviewed three factors affecting the polymorphic phase transformation in ZTA femoral heads (i.e., the initial *m*-ZrO_2_ content, surface stress, and metallic stains). Each of these is inexorably connected to the hydrothermal effect as well. Individually or in combination, they trigger destabilization of the *t*-ZrO_2_ phase. Mechanistically, the size of the zirconia domains regulates the initial amount of the monoclinic phase, while tensile stresses enhance existing nuclei growth, and metal stains accelerate nucleation of *m*-ZrO_2_ sites. There concomitant contributions resulted in the phenomenological plot shown in Figure 1. However, tensile stresses exhibited a fundamentally different kinetic behavior (i.e., an enhanced Avrami slope) when compared to data from retrievals. Moreover, the development of local residual stresses in the ZTA microstructure should be a *consequence* of the polymorphic transformation rather than a prerequisite. This assertion can be experimentally justified by observing residual stresses using CL analyses for off-stoichiometrically drifting sites [33,34,35]. This technique involves probing the sample using a low-voltage electron beam with nanometer-scale resolution (i.e., spatial resolution two orders of magnitude higher than the Raman probe) to concurrently reveal local off-stoichiometry and lattice stresses in alumina-based bioceramics. At the molecular scale, local stoichiometry and stress are linked and can be analyzed by monitoring the luminescence emission for oxygen vacancies, *V_o_*, in the Al_2_O_3_ lattice. The *V_o_* sites emit a doublet, which corresponds to oxygen vacancy sites charged with one or two electrons [36]. The photon intensity emitted by electron-irradiated samples (i.e., the area subtended by the *V_o_* doublet) is proportional to the number of defective sites lying within the probe (i.e., stronger intensities correlate to greater surface off-stoichiometry). The wavelength of the emission relates the stress field to the vacancy concentration; and this has been precisely calibrated using both single-crystal sapphire and polycrystalline alumina samples [31,32,33]. Figure 11a shows a CL assessment of the stress field in the vicinity of metastable zirconia domains in pristine BIOLOX^®^*delta* ZTA femoral heads and after they have been subjected to increasing exposures in the autoclave at 121 °C (i.e., for 5~300 h). The resulting CL maps for *V_o_* sites clearly show an increase in tensile residual stress within the alumina matrix surrounding the zirconia domains (emphasized in black color). The plots shown in Figure 11b were generated by concurrently observing the off-stoichiometric drift within the alumina matrix (i.e., the intensity of the CL emission from *V_o_* sites) and the polymorphic transformation (i.e., by Raman spectroscopy).

These results reveal that the oxygen vacancy concentration within the alumina lattice sharply increases with autoclave exposure and saturates after about 50 h (cf., an abrupt change in the slope of the oxygen-vacancy curve of Figure 11b). The threshold of 50 h exposure also corresponds to a steep rise in *m*-ZrO_2_ suggesting a link between the off-stoichiometry of the alumina lattice and the destabilization of zirconia dispersoids. Oxygen-vacancy formation occurs due to dehydroxylation of the alumina surface, which in turn is caused by thermal activation of the O-H bonds [37,38]. Note that this phenomenon is peculiar to the alumina lattice. Conversely, the zirconia hydroxylated surface is quite stable; it hardly loses oxygen to form new vacancy sites. The zirconia dispersoids are partially stabilized in their tetragonal polymorph by doping with sub-valent yttrium (Y^3+^), which substitutes for zirconium (Zr^4+^) in the lattice. This substitution creates oxygen vacancies in order to maintain charge balance in the lattice; and it is the main reason why the tetragonal polymorph can be partially stabilized at room temperature [39]. The data in Figure 11 suggest that alumina plays a self-sacrificing role in protecting the metastable *t*-ZrO_2_ domains from prematurely transforming to their stable *m*-ZrO_2_ form. However, as soon as dehydroxylation of the alumina lattice slows (i.e., after ~50 h exposure), pre-existing oxygen vacancies in the tetragonal zirconia lattice are progressively annihilated which triggers their transformation. Additionally, the observed tensile stress in the alumina lattice actually serves to counterbalance the compressive stress of the constrained zirconia domains after their volumetric expansion into the monoclinic polymorph.

**Figure 12:**
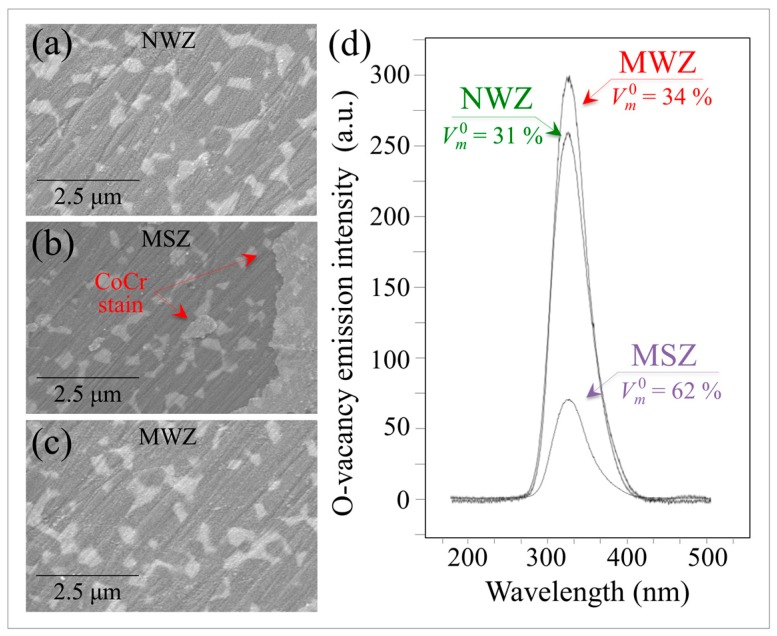
Scanning electron micrographs from the: NWZ (**a**); MSZ (**b**); and MWZ (**c**) of a CoCr metal-contaminated BIOLOX^®^*delta* retrieval. This head was in vivo only for a short time against a polyethylene liner. (**d**) Off-stoichiometry drifts measured by the average CL emissions for *V_O_* in different zones of the retrieval (cf. zones of spectral analysis and monoclinic fractions located by labels).

Now, with regards to the molecular scale interaction of metallic stains, it should be noted that the chemical elements comprising all metal stains in hip arthroplasty (i.e., Ti, Fe, Co and Cr) belong to period 4 of the Table of Elements (i.e., the first 3*d* orbital). Their ability to adopt multiple oxidation states and to form complexes through utilization of 3*d* and 4*s* electrons is well known [40]. These peculiar characteristics increase the concentration of reactants at their surfaces while concurrently weakening bonds in catalytically reacting molecules. Figure 12a–c shows scanning electron micrographs from the NWZ, MSZ, and MWZ of a CoCr metal-contaminated BIOLOX^®^*delta* retrieval, respectively. This head was in vivo only for a short time against a polyethylene liner.

**Figure 13:**
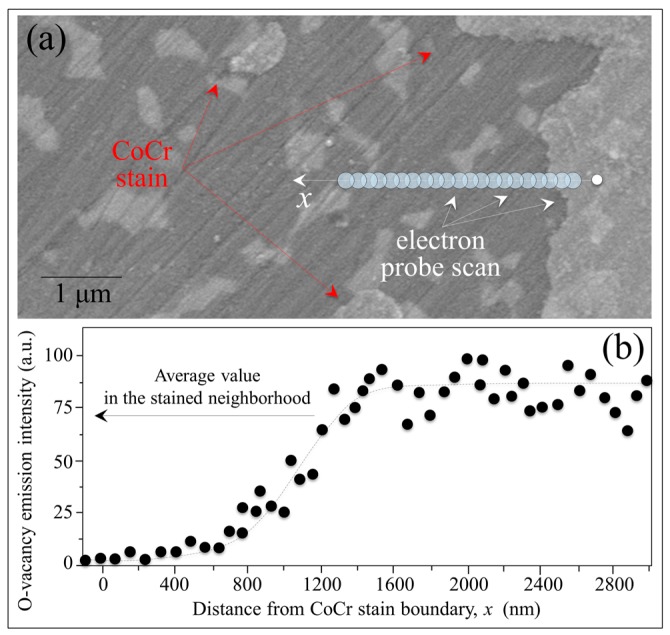
(**a**) Scanning electron image and location of CL scan as a function of distance, *x*, from a CoCr-contaminated zone for a hydrothermally-treated ZTA head; and (**b**) the related *V_O_* emission profile.

In comparing the three micrographs, note that there is no significant wear damage or other signs of mechanical interaction at the ZTA’s surface. Machining lines are equally observed in each micrograph. Although the different zones appear indistinguishable except for the presence of the CoCr stain in the MSZ (cf. arrows in Figure 12b), their monoclinic contents clearly differ (cf. labels in the inset to Figure 12d). The MSZ area contained a twofold higher local amount of *m*-ZrO_2_ as compared to both the MWZ and NWZ. The MWZ showed a slightly higher amount of the monoclinic phase when compared to the NWZ. These observations suggest that the driving force for the polymorphic transformation is predominantly chemical in nature. It is accelerated by the presence of a transition metal, although possibly exacerbated by enhanced frictional effects. Monitoring of the oxygen vacancy population by CL in different Al_2_O_3_ zones revealed that the highest concentration was in the MWZ. The NWZ also exhibited some off-stoichiometry drift, but the Al_2_O_3_ lattice in the MSZ was close to stoichiometric. The average CL emissions for *V_O_* in different zones of the retrieval are shown in Figure 12d. These data suggest that the CoCr metal stain effectively impedes dehydroxylation and oxygen-vacancy formation in the Al_2_O_3_ lattice. Additional evidence that metal stains annihilate oxygen vacancies is provided by the CL line scan of Figure 13a. This graph provides CL results as a function of distance, *x*, from a CoCr-contaminated zone for a hydrothermally-treated ZTA head. The *V_o_* emission profile (Figure 13b) essentially showed the annihilation of oxygen off-stoichiometry for a distance *x* ~ 400 nm. This was followed by its exponential rise to a saturated level similar to the MSZ *V_o_* concentration shown in Figure 12b.

When water from the hydrothermal environment binds to transition metal molecules, several proton-coupled electron-transfer events occur that oxidize the metal-water complex. Electrons are stripped away and participate in the process of water reduction. All these metallic elements present the same basic characteristics although they exhibit different levels of efficiency in splitting water. The half-filled *d*-orbitals facilitate both the binding of water and the release of molecular oxygen and hydrogen. It is also known that chromium atoms can easily diffuse along the Al_2_O_3_ surface, moving from one three-coordinated Al site to another since their activation energy for self-diffusion is lower than for Al atoms [41]. Chromium ions form strong Cr-H bonds with water molecules. They trigger autocatalytic dissociation of water due to splitting of the polar molecule to form Cr-H bonds. This behavior is consistent with *V_o_* annihilation in the Al_2_O_3_ lattice, both for in vivo and in vitro CoCr stains (cf. CL spectrum in Figure 12b and the disappearance of emissions from oxygen-vacant sites in the profile of Figure 13b), It is therefore proposed that Cr^3+^ separates from the stain and enters Al^3+^ sites close to oxygen vacancies while free oxygen from the split water molecules fills pre-existing vacancies. Concurrently, strong Cr-H bonds form with available hydrogen to impede further surface dehydroxylation. Accordingly, new oxygen vacancies form in the Al(Cr)O_3_ lattice close to the stained area. In this stoichiometrically “locked” environment, the propensity of the Al_2_O_3_ lattice to release oxygen and form stable water molecules or incorporate free oxygen is conspicuously eliminated. This effect fully exposes the metastable *t*-ZrO_2_ lattice to free oxygen absorption, resulting in its destabilization and transformation to the *m*-ZrO_2_ polymorph.

It is also well known that iron oxide is an effective catalyst for the oxidation of water; and its catalytic efficiency increases 50~80 times in the presence of alumina. This enhancement stems from the interaction of Fe with Al. Their combination alters the surface redox processes favoring the production of strong oxidants during water decomposition [42]. On the ZTA surface, iron ions in contact with the alumina matrix favor a radical water decomposition mechanism (series of 1e^−^ transfer steps) over a non-radical mechanisms (2e^−^ transfer step), leading to enhanced ^•^OH production. Lim et al. [43] postulated that alumina (a Lewis acid) expedites the reduction of Fe (III) in presence of water because it attracts electrons from iron thereby raising the oxidation potential of the Fe (III) center.

With regards to the titanium stain, it readily oxidizes to TiO_2_ when exposed to a hydrothermal environment. The resulting oxide layer has an ability to form electron-hole pairs. Electrons are released at the material’s surface and are scavenged by molecular oxygen producing superoxide radicals. While this reaction is competitive with electron-hole recombination, it also initiates a free-radical chain by forming hydroxyl radicals, ^•^OH [44,45].

**Figure 14:**
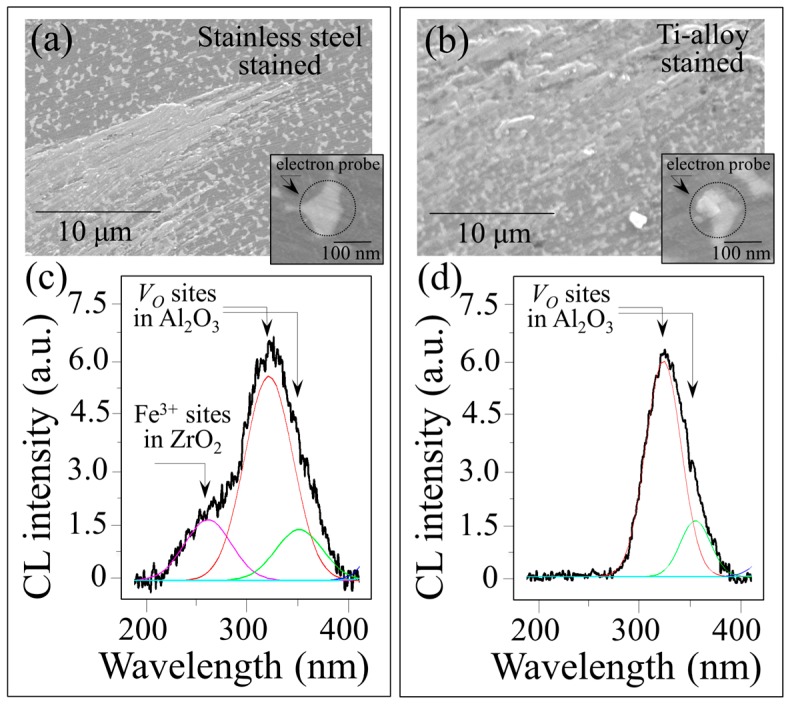
Scanning electron micrographs of Fe- (**a**) and Ti-contaminated (**b**) ZTA surfaces after an in vitro hydrothermal cycle of 200 h. Corresponding CL spectra are shown in (**c**,**d**), respectively, as collected at the spots depicted in the insets to each micrograph.

CL studies in the vicinity of Fe (from stainless steel) and Ti contaminants confirmed the annihilation of *V_o_* sites. However, there were differences in the CL spectra for these metallic stains. Figure 14 compares CL spectra collected for Fe-(a) and Ti-contaminated (b) ZTA surfaces after an in vitro hydrothermal cycle of 200 h. The CL spectra shown in Figure 14c,d were collected at the spots depicted in the insets of Figure 14a,b, respectively. The results for iron suggest that a fraction of Fe^3+^ ions penetrated the zirconia lattice substitutional for Zr^4+^. This was confirmed by a sub-band emitted at ~455 nm in the CL spectrum shown in Figure 14c. Conversely, there were no CL bands in the Ti-contaminated spectra that suggested Ti^4+^ substitution into the ZrO_2_ lattice (cf. Figure 14d). The CL spectra from both Fe- and Ti-contaminated ZTA presented similarly weak emissions from oxygen vacancies in the Al_2_O_3_ lattice, which confirmed the process of vacancy annihilation described above for CoCr contamination. In principle, dilute amounts of sub-valent Fe^3+^ substitutational located on Zr^4+^ sites could generate vacancies and trigger destabilization of the tetragonal lattice [46]. However, the enhanced kinetics detected by Raman spectroscopy suggests that this phenomenon did not delay the onset of the polymorphic transformation.

**Figure 15:**
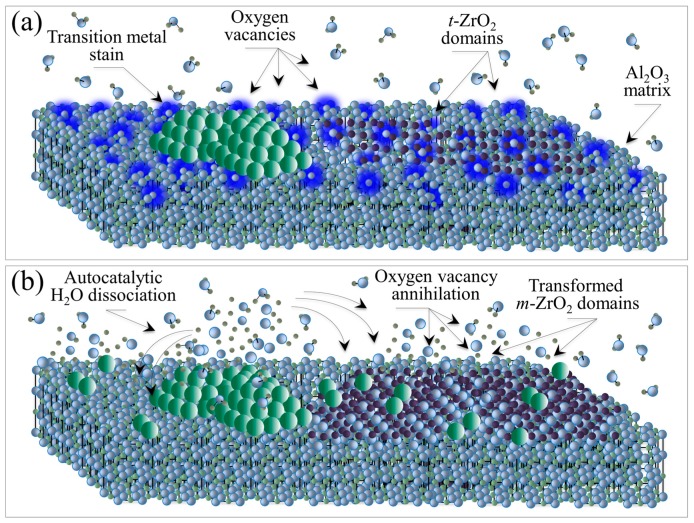
(**a**) ZTA destabilization due metallic contamination at the composite’s surface involving oxygen vacancies in both alumina and zirconia lattices; and (**b**) off-stoichiometry events leading to polymorphic transformation in the zirconia phase.

Despite having valences and ionic radii smaller than Zr^3+^, neither Ti, nor Co or Cr affected the CL spectrum of ZrO_2_, thereby ruling out any effect of frictionally induced mechanical alloying. In other words, a reduced concentration of oxygen vacancies in the ZrO_2_ lattice was the key factor in the enhanced transformation kinetics. A schematic molecular model for the surface destabilization of ZTA contaminated with transition metals under frictionally loading within a hydrothermal environment is shown in Figure 15.

In summary, the proposed new model for ZTA destabilization is based on metallic contamination at the composite’s surface and oxygen vacancies in both its alumina (i.e., dehydroxylation) [47] and zirconia (i.e., yttria doping) [48] lattices (Figure 15a). There are three successive off-stoichiometry events (Figure 15b): (i) autocatalytic dissociation of water at the metal surface; (ii) oxygen vacancy annihilation in the Al_2_O_3_ matrix until exhaustion; and (iii) oxygen vacancy annihilation in the *t*-ZrO_2_ domains followed by their transformation into *m*-ZrO_2_. All transition metals that frequently contaminate ZTA femoral heads in vivo detrimentally contribute to its surface destabilization. The contamination process, which induces off-stoichiometric drifts at the ZTA surface, is mainly chemically driven but enhanced by frictional effects and related surface charges. This new model explains the accelerated transformation kinetics; and it is in agreement with recently published X-ray photoemission spectroscopy data [49]. A quantitative expression to replace the traditionally used MAJ [7,21,22,23] equation has been developed and its derivation is provided in a separate publication [25].

## 4. Conclusions

This paper started with a review of the observed phenomenological discrepancies between surface instabilities observed for short- and medium-term BIOLOX^®^*delta* ZTA retrievals. Using ASTM F2345-03, the predicted transformation kinetics from in vitro exposed heads was several orders of magnitude slower than what was observed for time-matched actual retrievals. To investigate and rationalize this discrepancy, a series of in toto spectroscopic analyses were conducted using pristine and in vitro hydrothermally aged ZTA femoral heads and compared to actual in vivo retrievals. These analyses provided a more complete picture of the causes of ZTA surface instability. Accordingly, the key questions posed in the abstract of this paper can now be answered: (i) Why do in vitro hydrothermal treatments conspicuously fail to reproduce the in vivo environment? This is because current in vitro hydrothermal test only addresses one of several triggers contributing to ZTA surface degradation in vivo; (ii) Which microscopic phenomenon is preponderant in triggering the transformation in vivo? The chemical effect induced by transition metal contamination appeared as a key factor in accelerating the kinetics of the polymorphic transformation of the *t*-ZrO_2_ domains. However, coarsening of the ZTA microstructure which resulted in abnormally high monoclinic fractions in as-received components also played a fundamental role in accelerating the in vivo kinetics; (iii) What revisions to the present industry standards should be considered in order to obtain sound predictions of the ZTA transformation kinetics in vivo? A revision of ASTM F2345-03 should include an updated predictive equation along with changes to its testing protocol. The new equation will not only account for greater influence from the initial monoclinic fraction, *V_m_*^0^, but it will also factor in activation energy effects associated with metallic contaminants. The protocol of this in vitro standard should also be changed to include staining of ZTA samples with transition metals since they proved to be extremely detrimental to surface degradation. In reality, their presence on implanted femoral heads is almost unavoidable. Note also that femoral stems made of transition metals strongly contaminate ZTA heads at the trunnion-head interface due to high tensile stresses. Therefore, it is critical to properly assess the impact that chemically triggered phase instability may have on predicted stresses at the interface. Lastly, ISO 13356 needs to be modified in order to restrict the amount of allowable *m*-ZrO_2_ to ≤16 vol.% (i.e., the theoretical percolation limit) in order to limit the risk of accelerated phase instability, at both the surface and sub-surface of the ceramic.

## Figures and Tables

**Table 1:** List of the investigated retrievals, their respective year of manufacture, in vivo service lifetimes, and the reasons for removal during revision surgery.

Sample	Type	Manufactured Year	Time In Vivo (mo)	*V_m_* (%) MWZ	*V_m_* (%) NWZ	Metal Stain	Cause of Revision
1	CoP	2010	1.8	49	32	Yes	Dislocation
2	CoP	2009	3.5	43	34	No	Infection
3	CoC	2011	8.4	44	18	Yes	Dislocation
4*	CoC	2008	9.0	32	19	Yes	Septic mobilization
5	CoP	2010	9.2	37	31	No	Infection
6	CoC	2011	13.2	36	34	No	Infection
7	CoP	2012	14.8	35	27	No	Infection
8	CoC	2009	14.9	53	47	Yes	Dislocation
9	CoP	2008	14.9	51	40	Yes	Dislocation
10	CoC	2009	15.3	47	38	No	Infection
11	CoC	2009	16.0	60	51	Yes	Dislocation
12	CoP	2010	16.4	42	35	No	Thigh pain
13*	CoC	2006	20.2	48	40	Yes	Dislocation
14	CoC	2009	28.4	11	10	Yes	Septic mobilization
15	CoP	2009	34.7	55	46	Yes	Dislocation
16	CoP	2007	41.2	65	35	Yes	Dislocation
17	CoC	2009	55.5	44	41	No	Aseptic loosening
18	CoP	2009	66.7	51	37	No	Stem loosening
19*	CoC	2008	77.3	28	19	Yes	Aseptic mobilization
20	CoC	2009	83.4	57	41	Yes	Dislocation
21	CoC	2010	95.8	43	31	No	Aseptic loosening
22*	CoC	Unknown	106.5	36	13	Yes	Dislocation
